# Genetic and parasitological identification of *Trypanosoma evansi* infecting cattle in South Sulawesi, Indonesia

**DOI:** 10.14202/vetworld.2021.113-119

**Published:** 2021-01-15

**Authors:** Agus Setiawan, Wisnu Nurcahyo, Dwi Priyowidodo, Rina Tri Budiati, Desy Sylvia Ratna Susanti

**Affiliations:** 1 Department of Animal Quarantine, Indonesia Agricultural Quarantine Agency, Makassar, Indonesia; 2 Department of Parasitology, Faculty of Veterinary Medicine, Universitas Gadjah Mada, Yogyakarta, Indonesia; 3 Department of Veterinary Science, Faculty of Veterinary Medicine, Universitas Gadjah Mada, Yogyakarta, Indonesia

**Keywords:** cattle, internal transcribed spacer-2, Sulawesi, Surra, *Trypanosoma evansi*

## Abstract

**Background and Aim:**

Sulawesi is an Indonesian island located within the Wallacea region that contains a distinctive mix of Asian and Australasian species. This distinctiveness extends to parasites, including *Trypanosoma evansi*, the cause of surra. Surra has non-specific clinical signs such as anemia, anorexia, weight loss, drop in milk production, and reproductive disorders which cause economic losses. Due to the trade of livestock, surra has spread in Indonesia from one island to another. The aim of this study was to investigate the trypanosomes infecting cattle in South Sulawesi, using internal transcribed spacer (ITS2) ribosomal DNA (rDNA) sequencing.

**Materials and Methods:**

A total of 100 whole blood samples were collected from cattle in Makassar, South Sulawesi Province, Indonesia. All samples were tested using conventional parasitological methods (CPT), namely, thin blood smear, buffy coat smears, and polymerase chain reaction (PCR) testing. Positive PCR results were sequenced and phylogenetically analyzed.

**Results:**

Only one of the 100 samples was found to be positive with microscopic observation; however, PCR analysis revealed that 3% (3/100) of samples were positive. Sequencing identified the positive samples as *T. evansi*, China isolate (KU552344), with a homology of 99%. Two out of three sequences showed variations in ITS2 region.

**Conclusion:**

Based on CPT and molecular analysis, *T. evansi* isolates from infected cattle in South Sulawesi demonstrate genetic diversity of ITS2 sequences.

## Introduction

Surra is a disease caused by *Trypanosoma evansi* [1], affecting several vertebrate hosts. *T. evansi* entered Southeast Asia through livestock imported from India [2]. In Indonesia, surra was first reported in 1898. Since then, surra has rapidly spread throughout the Indonesian islands through livestock exchange, and cases of *T. evansi* infection have been reported in almost all Indonesian regions [3]. Of all known pathogenic trypanosomes, *T. evansi* has the widest host range, infecting both wildlife and domestic animals [4]. Notably, livestock trading on Sulawesi, the largest island in Wallacea region, had a profound impact on the spread of *T. evansi*. Sulawesi is a significant biodiversity hotspot, it has the highest number of mammals of which 83 of 132 species (63%) are endemic that support substantial undocumented parasite diversity [5,6]. In addition, Sulawesi’s position between the Asian and Australian continental is home to endemic parasites and likely to be relevant to the biogeography of trypanosomes, such as *T. evansi* [7,8].

Surra cases recur throughout the year in South Sulawesi and cause livestock mortality, while research on *T. evansi* in South Sulawesi has not provided sufficient data. The phylogenetic diversity of trypanosome in Sulawesi has been studied in *Trypanosoma theileri* and *Trypanosoma lewisi* in murine rodents. *T. theileri* clade is native to Sulawesi and *T. lewisi* clade invaded Sulawesi recently [9]. It is important to do the phylogenetic analysis of *T. evansi* in Sulawesi to differentiate between the native and introduced *T. evansi* to better understand which changes may be associated with adaptation to the new environment related to the pathogenesis.

In this study, we investigated the species of trypanosomes infecting cattle in South Sulawesi using internal transcribed spacer (ITS2) ribosomal DNA (rDNA) sequence analysis. Our data may inform the implementation of governmental prevention programs to control the spread of surra on Sulawesi Island.

## Materials and Methods

### Ethical approval

All procedures performed in this study were approved by the Ethical Clearance Committee of the Faculty of Veterinary Medicine, Universitas Gadjah Mada, Indonesia (clearance number 0131/EC-FKH/Int./2019).

### Study period, area, and sample collection

**Figure-1:**
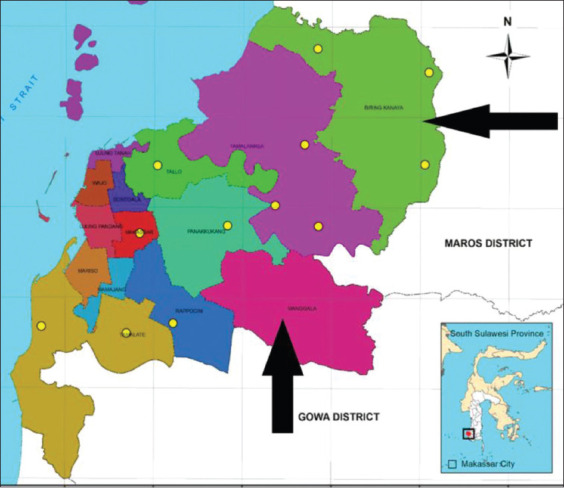
Map of Makassar, South Sulawesi. Location of sampling sites shown in black arrows (Source: Arif *et al.*, [8]).

This research was conducted for 5 months (October 2019 to February 2020) consisting of pre-research, samples collection and laboratory examination. Blood samples were taken from 100, stochastically chosen adult cattle in two geographically separated areas in Makassar (Figure-1). We obtained samples from Biringkanaya district that surrounded by rice fields and Manggala district that is close to the garbage dump. Blood (5 mL) was collected from each animal into a tube (OneMed, Indonesia) containing the anticoagulant ethylenediaminetetraacetic acid by jugular venipuncture and stored in refrigerated condition (4°C) until arrival at the laboratory. Thin blood smears were prepared from each sample a few seconds after blood collection. Approximately 75 mL of blood was moved into a capillary tube (Brand, Germany) and centrifuged for 5 min at 12,000 g. Next, the buffy coat was dropped onto an object-glass in preparation for buffy coat smears. All smears were dried, methanol-fixed, stained with Giemsa, and microscopically examined under 100× oil immersion.

### DNA extraction, polymerase chain reaction (PCR) amplification, and sequence analysis of rDNA ITS2

DNA was extracted from blood samples using the DNeasy^®^ blood DNA extraction kit (Qiagen, USA) following the manufacturer’s instructions; DNA was stored at −20°C until used. Forward (5’-CTC CTC GTG TGG TGC ATA TT-3’) and reverse (5’-GAA GCG TAC ACG AAA GAA GC-3’) primers for PCR amplification were designed based on *T. evansi* nucleotide sequence (accession number FJ416612) in the GenBank database using the primer3online program (http://bioinfo.ut.ee/primer3-0.4.0). PCR was carried out in a 25 μL reaction volume containing 12.5 μL of master mix (MyTaq™ Mix, Bioline), 5 μL DNA template, 1 μL (10 μmol/L) each of primer, and 5.5 μL ddH_2_O. The PCR amplification was performed as follows: An initial hold at 94°C for 4 min, then 35 cycles of denaturation at 94°C for 30 s, annealing at 57°C for 30 s, extension at 72°C for 30 s, and a final hold at 72°C for 5 min. PCR products were subjected to electrophoretic separation on 1% agarose gel at 100 volts for 30 min, stained with the FluoroVue™ Nucleic Acid Gel Staining reagent (SMOBIO Technology Inc., Taiwan), and visualized on an ultraviolet transilluminator. The positive samples were sequenced at First Base, Malaysia.

### Phylogenetic analysis

Positive PCR sequences were used to query the National Center for Biotechnology Information database using Basic Local Alignment Search Tool (BLAST) for identify sequence matches. Sample sequences were aligned against homologous sequences deposited in GenBank and analyzed using the ClustalW and MEGA (version X) software programs [10]. Phylogenetic trees were constructed based on the sequence data of the ITS2 rDNA regions of the samples and reference GenBank sequences. The analysis was performed using the neighbor-joining method and each branch point was evaluated with 1000 bootstrap replications. *Trypanosoma cruzi* (AF362827) was used to root the constructed tree.

## Results

### Morphology and PCR result

**Figure-2:**
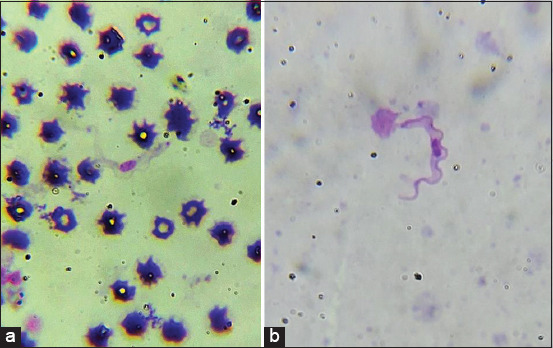
Morphology of *Trypanosoma evansi*: (a) Blood smear and (b) buffy coat smear, stained with Giemsa and observed under light microscopy; 1000× total magnification.

**Figure-3:**
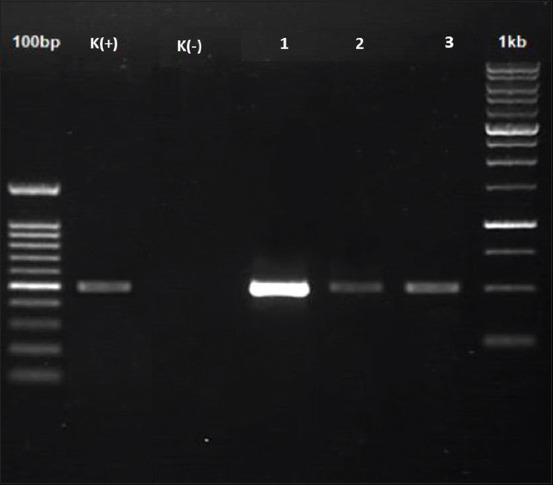
Polymerase chain reaction detection of *Trypanosoma evansi*, visualized as a positive band (508 bp) on 1% agarose gel. K(+): Positive control; K(−): Negative control; 1, 2, and 3: Positive samples.

One positive sample was found during the microscopic observation of 100 samples, which were assessed using both thin blood and buffy coat smears. Both smears of the positive sample showed the presence of trypanosomes (Figure-2) with the same morphology as described by Desquesnes *et al*. [11]. PCR analysis using ITS2 primers showed that 3 out of 100 samples (3%) were infected with trypanosome parasites; the 508 bp bands clearly indicated the presence of parasites in positive cattle blood samples (Figure-3).

### DNA sequence analysis

The ITS2 sample sequences were compared with other sequences from GenBank using BLAST. The positive sample sequences were revealed to share 99% similarity with *T. evansi* from some countries such as China, the Philippines, Thailand, Taiwan, Indonesia, Egypt, Colombia, and India. The sequences showed 98% similarity to *Trypanosoma equiperdum* and *Trypanosoma brucei brucei* and *Trypanosoma brucei gambiense*.

A subsequent analysis of variation between ITS2 samples and Guangdong China isolate (KU552344) revealed the presence of several mutations in ITS2 samples (Table-1). Categories of mutations were present; two instances of transverse mutation of samples AW4, two instances of transition mutations of samples AW12, one instance of transition mutations, and three instances of transverse mutations of samples AW45.

**Table-1:** Nucleotide variations and type of mutations between ITS2 samples and *T. evansi* from Guangdong China.

Species	Nucleotide sequence number						

	60	257	318	345	416	456	457
*T. evansi* Guangdong China	A	G	G	G	A	T	A
Trypanosoma_Makassar AW4	A	G	G	G	A	A	T
Trypanosoma_Makassar AW12	A	A	A	G	C	T	A
Trypanosoma_Makassar AW45	T	A	G	C	C	T	A
Type of mutation	TV	TS	TS	TV	TV	TS	TS

*Description: TV = Transverse mutation; TS = Transition mutation. ITS2= Internal transcribed spacer, *T. evansi* = *Trypanosoma evansi*

### Phylogenetic analysis

**Figure-4:**
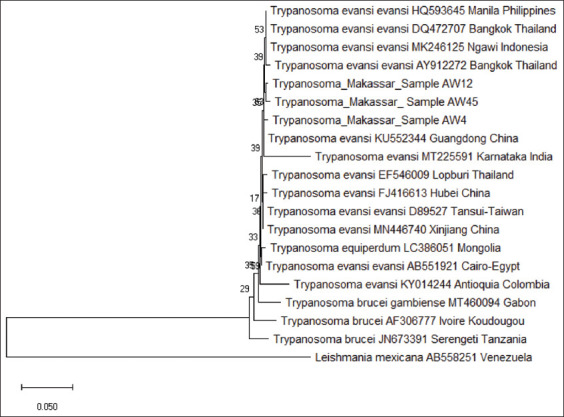
*Trypanosoma evansi* phylogenetic tree, based on ITS2 sequences, according to the neighbor-joining method, showing the position of Makassar strains.

The phylogenetic tree was generated with MEGA (version X) software using alignment data from ClustalW. The ITS2 partial sequences were compared to several intraspecies and interspecies using discontinuous megablast result. The phylogenetic tree (Figure-4) showed that the ITS2 samples AW4 had a short genetic distance to *T. evansi* Guangdong China (0.4%) and large genetic distance to *Leishmania mexicana* 56%, respectively (Table-2). The phylogenetic tree inferred from the ITS2 sequences (508 bp) illustrates the genetic diversity of the parasites. It showed that all samples were closely related to *T. evansi* from Guangdong China (KU552344) but two samples (AW12 and AW45) form a separate clade.

**Table-2:** Genetic distance of the ITS2 samples compared with intra- and interspecies.

Species	1	2	3	4	5	6	7	8	9	10	11	12	13	14	15	16	17	18	19	20
Trypanosoma_Makassar_Sample_AW4																				
Trypanosoma_Makassar_Sample_AW12	0.009																			
Trypanosoma_Makassar__Sample_AW45	0.011	0.007																		
*Trypanosoma evansi_*EF546009_Lopburi_Thailand	0.011	0.011	0.013																	
*Trypanosoma brucei gambiense*_MT460094_Gabon	0.033	0.033	0.035	0.031																
*Trypanosoma equiperdum*_LC386051_Mongolia	0.013	0.013	0.015	0.011	0.029															
*Trypanosoma evansi evansi*_MK246125_Ngawi_Indonesia	0.007	0.007	0.009	0.009	0.031	0.011														
*Trypanosoma evansi*_KU552344_Guangdong_China	0.004	0.004	0.007	0.007	0.029	0.009	0.002													
*Trypanosoma evansi evansi*_HQ593645_Manila_Philippines	0.007	0.007	0.009	0.009	0.031	0.011	0.000	0.002												
*Trypanosoma evansi evansi*_DQ472707_Bangkok_Thailand	0.007	0.007	0.009	0.009	0.031	0.011	0.000	0.002	0.000											
*Trypanosoma evansi evansi*_D89527_Tansui-Taiwan	0.007	0.007	0.009	0.004	0.027	0.007	0.004	0.002	0.004	0.004										
*Trypanosoma evansi*_MN446740_Xinjiang_China	0.007	0.007	0.009	0.004	0.027	0.007	0.004	0.002	0.004	0.004	0.000									
*Trypanosoma evansi evansi*_AY912272_Bangkok_Thailand	0.011	0.009	0.011	0.013	0.035	0.015	0.004	0.007	0.004	0.004	0.009	0.009								
*Trypanosoma evansi evansi*_AB551921_Cairo-Egypt	0.009	0.009	0.011	0.007	0.024	0.004	0.007	0.004	0.007	0.007	0.002	0.002	0.011							
*Trypanosoma evansi*_FJ416613_Hubei_China	0.011	0.011	0.013	0.009	0.031	0.011	0.009	0.007	0.009	0.009	0.004	0.004	0.013	0.007						
*Trypanosoma evansi*_KY014244_Antioquia_Colombia	0.038	0.038	0.040	0.035	0.055	0.035	0.035	0.033	0.035	0.035	0.031	0.031	0.040	0.031	0.035					
*Trypanosoma brucei*_AF306777_Ivoire_Koudougou	0.038	0.038	0.035	0.035	0.053	0.033	0.035	0.033	0.035	0.035	0.031	0.031	0.040	0.029	0.035	0.060				
*Trypanosoma brucei*_JN673391_Serengeti_Tanzania	0.038	0.038	0.040	0.035	0.053	0.033	0.035	0.033	0.035	0.035	0.031	0.031	0.040	0.029	0.035	0.060	0.053			
*Trypanosoma evansi*_MT225591_Karnataka_India	0.051	0.051	0.053	0.053	0.075	0.055	0.049	0.046	0.049	0.049	0.049	0.049	0.053	0.051	0.053	0.077	0.075	0.080		
*Leishmania mexicana*_AB558251_Venezuela	0.562	0.560	0.558	0.558	0.560	0.558	0.558	0.560	0.558	0.558	0.560	0.560	0.555	0.558	0.562	0.564	0.558	0.558	0.577	

ITS2=Internal transcribed spacer

## Discussion

Detection of trypanosome infection is usually performed by microscopic observation of the parasite in a Giemsa-stained blood smear. However, the parasite cannot always be detected by this method, even when animals display clinical symptoms [12]. Further, low-level parasitemia makes it impossible to detect and differentiate the morphology of parasites using Giemsa-stained smears [13]. In this study, only one positive sample (out of 100 total samples) was found through microscopic examination with both blood and buffy coat smears. This is similar to other reports from the literature. For example, Ahmadi *et al*. [14] detected four positive samples out of 117 blood samples using the Giemsa staining method. Another previous study reported that no positive samples were found out of 113 blood samples screened by microscopic examination, and seven positive samples were obtained by PCR [12]. Our data, taken together with reports from the literature, indicate that while microscopic examination of blood smears is the most commonly used method for the diagnosis of trypanosome infection, it is not suitable for epidemiological studies. Conventional parasitological method (CPT) is specific but not sensitive enough for efficient and widespread screening.

Newer molecular detection methods provide increased sensitivity. Using PCR, we demonstrated that 3 out of 100 samples (3%) were positive for *T. evansi*. As expected, the PCR method was able to detect a higher number of positive samples than CPT. In fact, other epidemiological studies indicate that PCR detection is more sensitive than hematocrit centrifugation technique [15]. The advantages of PCR as a diagnostic method have been further demonstrated during the chronic phase of trypanosome infection [16]. The sensitivity of this method is high: The threshold of detection is 1-20 parasite/mL of blood [17]. Our data confirm that PCR is the superior diagnostic method for trypanosomiasis.

Nucleotide changes in ITS2 samples as compared to ITS2 of *T. evansi* from Guangdong China impact on the ribosome biogenesis. ITS2 is a specific site of mutagenesis [18]. ITS2 as a scaffold to mediate topological rearrangement or as a timer to prevent premature folding that is critical in ribosome biogenesis. During this process, the 5.8S-ITS2-25S complex can rapidly fold in which the ITS2 folding could bring 5.8S and 25s pre-rRNA into close vicinity, thus facilitating hybridization [19] so that any sequence change could trigger conformational switches [20]. Mutation of sequence ITS2 samples may lead to the mutation of *T. evansi* in South Sulawesi. Genetic data, especially rDNA sequences, are critical for phylogenetic analysis, evolutionary process evaluation, and the determination of taxonomic identities of trypanosomes [21]. The ITS region consists of two regions (ITS1 and ITS2) that are located between the repeating array of nuclear *18S, 5.8S*, and *28S rRNA* genes [22]. Due to these features, the ITS region has been proven to be a useful tool in identifying both intra- and interspecific variability [23].

Many published reports have detailed the utility of ITS2 sequences in the phylogenetic study of trypanosomatids. It has been shown that ITS2 sequences are more useful for performing interspecific comparisons: ITS2 sequences are more variable than the ITS1 and the 5.8S rDNA sequences [24]. Khuchareontaworn *et al*. [23] also reported that the ITS2 region was informative for characterizing the genetic diversity of water buffalo trypanosomatids (four groups were detected). Similarly, Amer *et al*. [25] reported that analysis of the ITS region is very useful for elucidating the genetic diversity of *T. evansi* population of Egyptian camels. In another report, the phylogenetic tree inferred from ITS2 sequences clearly showed the genetic diversity of *T. evansi* infected camels in Sudan [26]. Genetic variations of sequence ITS2 samples may cause by the adaptation of *T. evansi* to the new environments, proven by the difference of sequence samples between Biringkanaya district and Manggala district. These data also suggest that *T. evansi* from Biringkanaya district, which is close to the garbage dump, adapt further to the environment or another possibility is that *T. evansi* is native to Sulawesi.

This is the first study using ITS2 sequences in the analysis of Sulawesi’s *T. evans*i isolates. Our data confirm previous observations of *T. evansi* genetic diversity [23] from two geographically separated areas in Makassar, South Sulawesi. Our phylogenetic and genetic analysis suggests that all sequences of ITS2 samples were most closely related to *T. evansi* from Guangdong, China but AW12 and AW45 samples that obtained from Manggala district display characteristics indicative of them being clade that is native to Sulawesi. Conversely, AW4 sample obtained from Biringkanaya district displays characteristic indicative of an introduced *T. evansi*. Notably, transportation of cattle from different islands in Indonesia may have an impact on the heterogeneity and dynamics of *T. evansi* populations in Sulawesi, Indonesia. The high degree of genetic variability with *T. evansi* population is most likely related to its capacity for rapid adaptation to different hosts and environments.

## Conclusion

Based on the microscopic parasitological methods and genetic analysis, the trypanosomes infecting cattle in Makassar, South Sulawesi, are *T. evansi*. In addition, phylogenetic analysis using ITS2 sequences identified two branching groups of *T. evansi*. This report provides preliminary data for studying the genetic diversity and dynamics of *T. evansi* in Indonesian livestock. This study will provide recommendations for future work to identify the distinct species of *T. evansi* in Sulawesi.

## Authors’ Contributions

AS, WN, and DP conceptualized the research, drafted the manuscript, prepared, and edited the manuscript according to the title. AS, RTB, and DSRS performed the research, analyzed the data, collected the literature, edited the manuscript, and finalized the manuscript. All authors read and approved the final manuscript.
